# Lorentz force induced shear waves for magnetic resonance elastography applications

**DOI:** 10.1038/s41598-021-91895-9

**Published:** 2021-06-17

**Authors:** Guillaume Flé, Guillaume Gilbert, Pol Grasland-Mongrain, Guy Cloutier

**Affiliations:** 1 Laboratory of Biorheology and Medical Ultrasonics, University of Montreal Hospital Research Center (CRCHUM), Montreal, QC H2X 0A9 Canada; 2 Institute of Biomedical Engineering, University of Montreal, Montreal, QC H3C 3J7 Canada; 3 MR Clinical Science, Philips Healthcare Canada, Markham, ON L6C 2S3 Canada; 4 Department of Radiology, Radio-Oncology and Nuclear Medicine, University of Montreal, Montreal, QC H3T 1J4 Canada; 5 Laboratory of Physics, ENS of Lyon, UMR 5672, Lyon, France

**Keywords:** Imaging, Diagnostic markers, Biomedical engineering, Techniques and instrumentation

## Abstract

Quantitative mechanical properties of biological tissues can be mapped using the shear wave elastography technique. This technology has demonstrated a great potential in various organs but shows a limit due to wave attenuation in biological tissues. An option to overcome the inherent loss in shear wave magnitude along the propagation pathway may be to stimulate tissues closer to regions of interest using alternative motion generation techniques. The present study investigated the feasibility of generating shear waves by applying a Lorentz force directly to tissue mimicking samples for magnetic resonance elastography applications. This was done by combining an electrical current with the strong magnetic field of a clinical MRI scanner. The Local Frequency Estimation method was used to assess the real value of the shear modulus of tested phantoms from Lorentz force induced motion. Finite elements modeling of reported experiments showed a consistent behavior but featured wavelengths larger than measured ones. Results suggest the feasibility of a magnetic resonance elastography technique based on the Lorentz force to produce an shear wave source.

## Introduction

Stiffness difference between healthy and abnormal biological tissues has long been put to use in the form of manual palpation to diagnose potential diseases. Although helpful to roughly estimate tissue stiffness, more precise and quantitative stiffness assessments can be reached using modern techniques such as shear wave elastography imaging^1–4^. This method relies on the propagation of shear waves of which the speed is proportional to the square root of the shear modulus of the medium^5^ in linearly elastic, homogenous, isotropic and infinite solids. Shear wave elastography requires to (1) induce shear waves within the sample, (2) track local displacements propagating as a shear wave, and (3) compute mechanical parameters from displacement images. Although some imaging techniques have been proposed to track shear waves, such as optical coherence tomography^6^ or a high speed camera^7^, main imaging methods for elastography remain ultrasound^8^ and magnetic resonance imaging (MRI)^9^. In MRI, computation of viscoelastic parameters has been performed with different algorithms, such as local frequency estimation (LFE)^10^, algebraic inversion of the equation of wave propagation^11–13^, identification methods using finite elements^14^, and temporal cross-correlation^15,16^.

In terms of shear wave generation, external actuators placed on the surface of the imaged tissue are mostly used in magnetic resonance elastography (MRE). In such cases, elastic waves propagate, and attenuate, from the surface to the region of interest while being tracked by the scanner. Pneumatic actuators have been widely used in MRE and have shown versatility through applications to liver^17^, brain^18^, heart^19^, and lung^20^. In this system, air pulses are generated by an active driver outside the MR room and routed through a plastic tube to a passive transducer attached to the patient. The setup is simple and handles well mono-frequency waveforms in the range of ~ 40 Hz to ~ 110 Hz. However, the uncontrolled acoustic response of pulsed air along the connection tube may render multi-frequency MRE difficult. Alternatively, a rigid rod has been attached directly to the pulsing active driver on one end and to a pad tied to the patient on the other end, which allowed for multi-frequency MRE^21–23^. Other successful surface actuation apparatus have been reported, such as piezoelectric^24^ and Lorentz coil drivers^25^ where the magnetic field of the MR scanner and the current injected in the coil combine to generate vibrations. So far, the presented solutions to produce displacement fields have in common the decrease of motion deflection as the driving frequency increases. This feature along with the inherent damping behavior of viscoelastic tissues limit in-vivo applications to low frequencies. To circumvent this limitation, mechanical devices relying on the centrifugal force have been presented, such as the “gravitational transducer”^26^. This system is made of a mass rotating about an axis of which the spinning frequency is driven by a motor outside the MR room. The centrifugal force exhibited in this type of actuator allows to increase the vibration amplitude by increasing the rotation frequency. This is an elegant solution to elicit displacement fields of sufficient amplitude at high frequencies within a reasonable distance from the surface actuator. However, challenges may remain at deep locations due to attenuation.

Instead of attempting to produce motion fields likely to reach the region of interest from the surface, an alternative may be to vibrate tissues closer to the targeted area thus shortening the propagation pathway and reducing the related wave damping. Ultrasound radiation pressure sources have been presented in that regard^27^ but carry practical implementation difficulties in the context of MR imaging. Intrinsic MRE, consisting in capitalizing on the natural pulsation of arteries as a motion source, requires no external actuator and has shown great potential at heartbeat frequencies^16,28–30^. Alternatively, the Lorentz force has been proposed for direct application of a motion (as opposed to Lorentz coils) to tissue mimicking phantoms by Grasland-Mongrain et al.^31,32^. They showed the possibility of producing shear waves within a conductive medium using the Lorentz force produced by a permanent portable magnet and external electrodes or a magnetic stimulation device, and an ultrasonic scanner for detection^31–33^.

Here, we hypothesized that a similar configuration could be used as a remote mechanism for shear wave induction in MRE by taking advantage of the strong magnetic field of MR scanners. Mechanical displacements of electromagnetic origin have been observed in soft solids using MRI^34^ but to our knowledge, no study has reported the use of the Lorentz force to produce in situ displacements in the context of MR elastography. In the present work, we propose to evaluate the feasibility of generating and detecting shear waves from a Lorentz force source within tissue mimicking samples. A specific MR compatible experimental setup was designed to isolate the Lorentz force contribution of the measured displacement field. Finally, preliminary conclusions on stiffness reconstructions from Lorentz force induced motion using the well-established LFE method are reported.

## Method

The proposed method consists in producing a Lorentz force density in tissue mimicking gels (phantoms) which in turn induces shear waves that are recorded using an MR clinical system and processed to retrieve elasticity information. The expression of the Lorentz force density [N.m^-3^] is given by

1 $${\varvec{f}} = {\varvec{j}} \times {\varvec{B}}$$

where ${\varvec{j}}$ is the current density [A m^−2^] and ${\varvec{B}}$ the magnetic field [T]^35^. Here, the strong homogeneous magnetic field of the MR scanner and externally applied currents were used to produce the Lorentz force.

### Experimental setup

**Figure 1:**
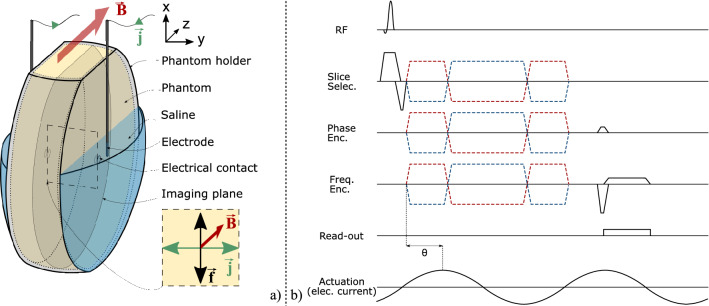
(**a**) Experimental setup. ${\varvec{B}}$ is the static magnetic field along the z axis produced by the MRI system.${\varvec{j}}$ is the harmonic current density applied to the phantom using external electrodes plunged into the saline baths and with a main oscillating direction along the y axis. ${\varvec{f}}$ is the Lorentz force produced within the phantom from the combination of **B** and **j**, and with a main oscillating direction along the x axis. For clarity, the main directions of ${\varvec{j}}$ and ${\varvec{f}}$ are represented and zoomed in the dashed cross-section. In the first experiment, electrical currents of 60 Hz and 90 Hz frequencies and of 137.5 mA peak-to-peak intensity were used. Phantom A was homogeneous and doped with NaCl to be electrically conductive. See Table 1 for phantom contents and Fig. 5 for details of the second experiment. (**b**) MRE pulse sequence diagram. MEGs of opposite polarities are indicated by the red and blue dashed lines. θ represents the phase offset between actuation and MEGs. MEGs along different directions (slice selection, phase encoding, and read-out) are successively activated in separate acquisitions.

In all the experiments reported in the present manuscript, agar phantoms were poured into a 3D printed mould and crossed by an electrical current ${\varvec{j}}$ and a magnetic field ${\varvec{B}}$ (Fig. 1a). Details about the different phantoms used are given in the paragraphs below and in Table 1. Saline baths (20%w) were designed on the two sides of the phantom holder to make the electrical contact between the phantom and external electrodes. This permitted to avoid mechanical contacts between electrodes and the phantom, thus allowing to isolate the Lorentz force density as the source of motion (i.e., avoid artefact movements). The electrical current was brought by means of electrodes (copper wires) plunged into the saline. Wires were placed parallel to the static magnetic field direction from the end of the bore to the phantom holder, and maintained fixed from above the phantom holder to the saline baths. Special care was taken to avoid mechanical contacts between wires and the rest of the setup to eliminate other movement artefacts. The phantom was placed in a 32-channel receive-only head coil in the bore of a 3 T MRI scanner (Achieva TX, Philips Healthcare, Best, Netherlands). The coil was positioned at the bore isocentre to minimise eddy currents and to facilitate the reproducibility of experiments. The phantom was oriented along the magnetic field (z axis, red arrow in Fig. 1a) and the current was mainly flowing along the *y* axis (green arrow in Fig. 1a) between the two electrodes so that the magnetic field and the current were perpendicular. The induced Lorentz force was oscillating along the *x* axis (black arrow in Fig. 1a) at the frequency of the current. A motion field was then produced with in-plane components parallel and perpendicular to the Lorentz force, i.e. along *x* and *z* axes. The electrical current was provided by a waveform generator (Agilent, 33250A 80 MHz), amplified (Brüel & Kjær, power amplifier type 2706) and routed through a 2-Ω shunt resistance in series with the phantom and the generator. Two sinusoidal waveforms of 60 Hz and 90 Hz were successively used in the experiments.

**Table 1:** Phantom properties.

Experiment #	Phantom	Agar (*w* %)	Saline (salt *w* % in volume water)
1	A	0.4	10
2	B	0.5	0.9
	C	0.7	0.9
	D	0.8	0.9
	E	1	0.9

### MRI acquisitions

For actuation and motion detection, current delivery to the phantom was synchronised with the motion encoding gradients (MEGs) of a 2D gradient echo sequence tailored to elastography experiments^36^ (Fig. 1b). The current generator was triggered by the MR scanner at the beginning of the MRE pulse sequence, in the same way as with other standard mechanical transducers. The phase accumulation experienced by spins undergoing harmonic actuation is given by:

2 $$\Phi ={\int }_{0}^{\tau }\gamma {\varvec{G}}\left(t\right)\bullet {\varvec{u}}\left({{\varvec{r}}}_{0},t\right)dt$$

where *γ* [rad s^−1^ T^−1^] is the sample’s gyromagnetic ratio, τ [s] the period of actuation, and ***G*** [T m^−1^] the motion encoding gradient amplitude. Generated motion was encoded along three orthogonal directions (anterior–posterior, right-left, foot-head). Parameters of the elastography sequence were: repetition time TR = 50 and 56 ms (adjusted to match a multiple of the shear wave vibration period), echo time TE = 15 and 20 ms, spatial resolution = 1.25 mm × 1.25 mm, slice thickness = 5 mm, elastography phases = 4, amplitude of the motion encoding gradients = 31 mT/m (highest value available to ensure maximum detection in the furthest regions from the motion source), and motion encoding over one cycle.

For displacement field measurements, two phase images were successively acquired with MEGs of opposite polarities. The second phase image was then subtracted from the first one using the exponential form of the complex MR signal to eliminate potential static phase offsets, thus producing a single phase difference image free of unwanted constant background. During the acquisition (whole TR) of both phase images, the generator was continuously delivering the same oscillating electrical current. The induced phase offset due to current injection in the phantom was then the same in these images and was consequently cancelled out in the phase difference operation. Additionally, receive coils were syntonised to the Larmor frequency of hydrogen’s proton and consequently show a low sensitivity to the low-frequency currents used in our study. To compute displacement maps from phase difference images, the encoding coefficient |*ξ*| in [rad  m^−1^] was computed using $\left|\xi \right|=\left|\frac{\gamma {G}_{0}\left(\mathit{sin}\left(\pi f\tau \right)-2sin\left(\frac{1}{2}\pi f\tau \right)\right)}{\pi f}\right|$, which approximates first moment nulling gradients and where *γ* [rad s^−1^ T^−1^] is the sample’s gyromagnetic ratio, *τ* [s] and *G*_*0*_ [T m^−1^] are the period and the amplitude of the MEG respectively, and *f* [Hz] is the excitation frequency^37^.

### Inverse problem and data processing

Once shear waves were produced and detected, the next step consisted in generating maps of viscoelastic parameters. The forward problem relating harmonic displacements to tissue mechanical properties is described by the Navier’s equation:

3 $$-\rho {\omega }^{2}{\varvec{u}}=\nabla \bullet G\nabla {\varvec{u}}+\nabla \left(\lambda +G\right)\nabla \bullet {\varvec{u}}$$

where *ρ* is the material’s density [kg m^−3^], *ω* the motion angular velocity [rad s^−1^], ***u*** the motion field [m], *λ* [N m^−2^] the first Lamé coefficient^38^ and *G* [N m^−2^] the complex shear modulus. In agar and agar-doped phantoms, the real part of the complex shear modulus (storage modulus) was shown to considerably dominate the imaginary part (loss modulus)^39,40^. The LFE, which ignores the loss modulus, was then chosen to retrieve first elasticity information from Lorentz force induced displacements in agar phantoms, given its availability and wide use in the MRE community. The LFE reconstructions could allow validating the working hypothesis to show the possibility of implementing the Lorentz force MRE method on a clinical scanner. Additionally, the LFE is known to be more robust against noise than other direct methods involving the estimation of second or higher order derivatives^11,13^.

The LFE consists in applying pairs of filters to the selected harmonic of the displacement data in the *k*-space. These filters are called log-normal quadrature filters and are generally centered on frequencies separated by one octave^41^. The ratio of displacements processed by each filter of one pair is equal to the local wave number. As the actuation frequency *ω* is known (*i.e.*, electrical current frequency), the wave number *k* can then be related to the local real value of the shear modulus in [N.m^-2^] using $\mu =\rho {\left(\frac{\omega }{k}\right)}^{2}$. It is equivalent to solving the Helmholtz equation $-\rho {\omega }^{2}{\varvec{u}}=\boldsymbol{ }\mu\Delta {\varvec{u}}$, which is a simplified version of the Navier’s equation under the assumption of tissue incompressibility, local homogeneity, and no shear wave attenuation. Shear modulus maps computed from displacements along the actuation direction (*x* axis) are reported in this study.

Prior to inversion, phase images were automatically unwrapped using a software embedded in the scanner (Resoundant Inc., Rochester, MN). Shear modulus images were then filtered to remove outliers using the smoothn Matlab function^42,43^. For displacement display (Fig. 3), a separate phase unwrapping procedure was used^44–46^.

### Experiment 1

To demonstrate the proposed method, two experimental conditions were designed, both consisting in generating shear waves in tissue mimicking gels. The first one intended to show the feasibility of generating shear waves with the Lorentz force under favourable conditions (i.e., an electrically conductive, soft and homogeneous phantom, termed phantom A in Table 1), and to observe them with the MRI scanner. Two sinusoidal electrical currents of 137.5 mA peak-to-peak with frequencies of 60 Hz and 90 Hz were respectively used. Such intensity is 69 times as large as values reported in electrical stimulation (ES) protocols performed on humans (*i*_*ES*_ = 2 mA peak-to-peak)^47^. Results are shown Fig. 3.

### Experiment 2

The purpose of the second experiment was to show that Lorentz force induced shear waves could be used to distinguish regions with different mechanical properties within the same heterogeneous structure using phantoms with different mechanical properties (termed B, C, D, and E in Table 1), and a less conductive medium corresponding to physiological conditions. To create the heterogeneous structure, phantom B was poured on top of phantom C in one phantom holder (Figs. 1a and 5A), and phantom D was poured on top of phantom E in another phantom holder (Figs. 1a and 5A). Sinusoidal currents of 62.22 mA peak-to-peak at frequencies of 60 Hz and 90 Hz were used, which corresponds to $33\times {i}_{ES}$.

### Simulations

**Figure 2:**
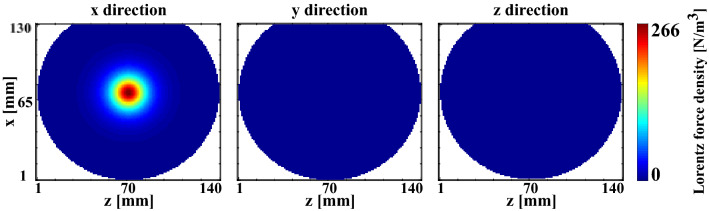
Simulated Lorentz force density maps in phantom A of the first experiment. Maps are located in the imaging plane of Fig. 1. The applied current was 137.5 mA peak-to-peak at a frequency of 90 Hz. A significant Lorentz force component is observed along the x axis, but it is negligible along the y and z axes.

A finite element physical model corresponding to the first experiment using phantom A was designed with Comsol 5.5 (COMSOL LiveLink with Matlab, Inc., Palo Alto, CA) to assess the displacement patterns obtained from a convolution of point-like sources, and to compare with experimental data. Simulations consisted in two steps, first computing the Lorentz force density distribution within the phantom, and second computing the wave field resulting from this Lorentz force density source term. To compute the Lorentz force density distribution, the following boundary value problem was solved using the electric current module:

$$\left\{\begin{array}{ll}\nabla \cdot \left(\sigma \nabla u\right)=0 \quad in \; the \; phantom\\ I={\int }_{in}\sigma \frac{\partial u}{\partial {\varvec{n}}}dS=-{\int }_{out}\sigma \frac{\partial u}{\partial {\varvec{n}}}dS \quad electrical \; contacts \; (in \;and \; out)\\ \nabla u\times n=0 \quad on \; electrical \; contacts\\ \sigma \frac{\partial u}{\partial {\varvec{n}}}=0 \quad on \; phantom \; walls \backslash electrical \; contacts\; in \; and\; out\end{array}\right.$$

where *u* is the electric potential [V], *σ* the electrical conductivity [S m^−1^], *I* the electrical current [A], *S* the surface areas [m^2^] of the electrical contact, and ***n*** is a unit vector perpendicular to the outer boundary. The externally applied electrical current *I* was modeled perpendicular to the phantom at the electrical contact location (Fig. 1a). The intensity and frequency of the current were the same as in the first experiment (60 Hz and 90 Hz, peak-to-peak amplitude of 137.5 mA). The phantom conductivity was homogeneous and set to 1 S/m as it impacts neither the current density distribution within the phantom nor its intensity, which are governed by the boundary conditions in this model. From the electric potential distribution *u*, the current density was computed using Ohm’s law: ${\varvec{J}}=-\sigma \nabla u$*.*. The Lorentz force density distribution in the phantom (Fig. 2) was then computed using Eq. (1):

$${\varvec{f}}=\left(\genfrac{}{}{0pt}{}{{j}_{x}}{\begin{array}{c}{j}_{y}\\ {j}_{z}\end{array}}\right)\times \left(\genfrac{}{}{0pt}{}{{B}_{x}}{\begin{array}{c}{B}_{y}\\ {B}_{z}\end{array}}\right)=\left(\genfrac{}{}{0pt}{}{{j}_{x}}{\begin{array}{c}{j}_{y}\\ {j}_{z}\end{array}}\right)\times \left(\genfrac{}{}{0pt}{}{0}{\begin{array}{c}0\\ {B}_{z}\end{array}}\right)$$

where $\left|{\varvec{B}}\right|=\left|{{\varvec{B}}}_{{\varvec{z}}}\right|=3 T$ is the magnetic field produced by the MR scanner.

To compute the displacement field resulting from the Lorentz force density distribution in the phantom, the following boundary value problem was solved using the solid mechanics module:

$$\bf \bf \bf \bf \bf \left\{\begin{array}{ll}-{\rho \omega }^{2}\varvec{u}-\nabla \bullet \left(G\nabla {\varvec{u}}\right)-\nabla \left(\left(K+\frac{4}{3}\mu \right)\nabla \bullet {\varvec{u}}\right)= \varvec{f} \quad in \; the \; phantom \\ \varvec{u}=0 \quad on \; phantom \; holde{r}^{^{\prime}}s \; walls\end{array}\right.$$

where ***u*** is the displacement field [m], *ρ* is the material’s density [kg m^-3^], *ω* is the pulsation [rad s^−1^], *G* is the complex shear modulus [N m^−2^], *K* is the bulk modulus [N m^−2^], and ***f*** is the Lorentz force density [N m^−3^]. The bulk modulus was set to 2.2 GPa (water). Although the approximation of no shear wave attenuation remains reasonable for agar phantoms in the calculation of the solution to the inverse problem, such approximation has large consequences on the calculated solution to the forward problem. The definition of a loss modulus was thus found necessary to account for the slight damping behavior of the phantom.

**Figure 3:**
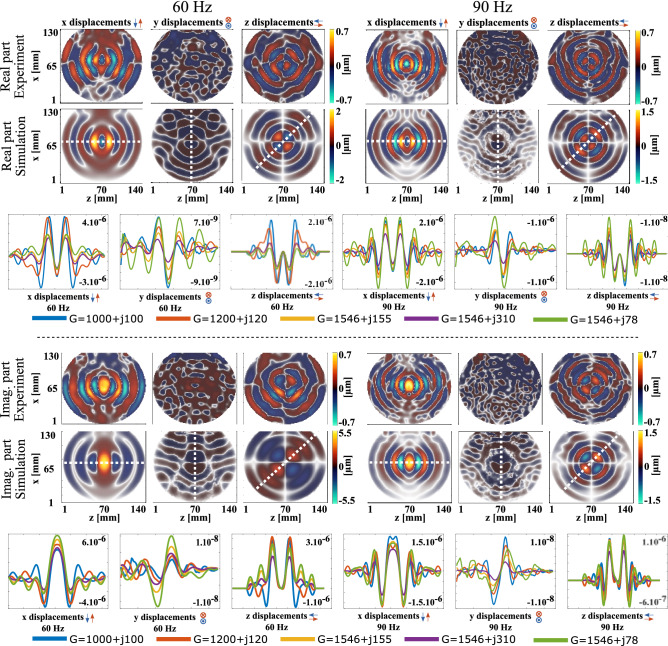
First experiment: Real and imaginary parts of the experimental and simulated complex displacement fields in phantom A along x, y, and z directions in the MR slice. The origin of elastic waves clearly appears at the centre of displacement maps. Images were acquired (experiment) and extracted (simulation) in the greyed-out sagittal plane located at the centre of the phantom, as indicated in Fig. 1 (“imaging plane” label). Two electrical currents were used with frequencies of 60 Hz and 90 Hz, and an intensity of 137.5 mA peak-to-peak in both experiment and simulation. The complex shear moduli used to obtain the displayed simulated displacement maps were: G_60Hz_ = 1546 + j155 Pa and G_90 Hz_ = 1768 + j177 Pa. Line profiles of the real and imaginary parts of displacements along the propagation directions (dashed white line) are plotted for each combination of storage and loss moduli described in Table 2. From these line profiles appear the diffraction patterns characteristic of body force actuation regardless of the complex shear moduli.

**Figure 4:**
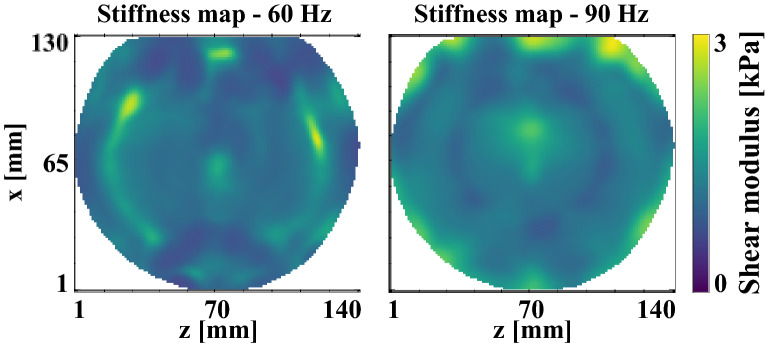
First experiment: shear storage modulus maps of homogeneous phantom A from x displacements with harmonic actuations of 60 Hz and 90 Hz and with an intensity of 137.5 mA peak-to-peak. Local maxima are assumed to be due to LFE limitations arising from the presence of the Lorentz force and wave reflections off boundaries. Images were acquired in the imaged plane indicated in Fig. 1a. The mean values over the entire field of views are $\mu {}_{60Hz}$=1546 ± 383 Pa and $\mu {}_{90Hz }=1768\pm 440$ Pa.

To fix the loss modulus *G*", the reported trend of the relationship between storage and loss moduli in agar phantoms was used. As aforementioned, the storage modulus was shown to dominate the loss modulus in agar and agar-doped phantoms. More specifically, around an order of magnitude difference can be observed between these two parameters^39,40^. The storage moduli at both frequencies were then fixed from the shear modulus distributions in Fig. 4, and the loss moduli were fixed from the above-mentioned *G*’–*G*” relationship. However, given the uncertainty on the phantom’s exact mechanical properties (variability in maps of Fig. 4 for *G*’ and approximated *G*”), the forward problem was solved multiple times using different sets of complex shear moduli. In the first simulation, *G*’ values at both frequencies were set to the mean values of the shear modulus distributions in Fig. 4: *G*’_*60 Hz*_ = 1546 Pa and *G*’_*90 Hz*_ = 1768 Pa. Loss moduli *G*" were set to 1/10th of the storage moduli: *G*”_*60 Hz*_ = 155 Pa and *G*”_*90 Hz*_ = 177 Pa. Results are shown in Fig. 3. In the second and third simulations, the same *G*’ values were used and *G*" values were varied and successively set to 1/5th and 1/20th of *G*’ values. In the fourth and fifth simulations, *G*’ values were changed towards the lower values in the shear modulus distributions in Fig. 4, and *G*" were set to 1/10th of *G*’ in each case. Results are shown in Fig. 3. All combinations of *G*’ and *G*" parameters are gathered in Table 2. This iterative framework intended to show that, despite uncertainties on the phantom’s exact mechanical characteristics, different possible storage and loss moduli do impact the shear wave propagation patterns and their wavelengths but do not question the Lorentz force density origin of the produced motion fields, which is what the simulation was meant to highlight.

**Table 2:** Complex shear moduli *G* = *G*’ + j*G*” in [Pa] used in the simulations.

Simulation #	Frequency	
	60 Hz	90 Hz
1	1546 + j155	1768 + j177
2	1546 + j310	1768 + j354
3	1546 + j78	1768 + j89
4	1200 + j120	1500 + j150
5	1000 + j100	1300 + j130

Regarding the geometry, side boundaries (phantom holder’s walls) were fixed while the upper boundary was left free to mimic the configuration of Fig. 1. Both calculations (electric potential and displacement field) were performed in the frequency domain. Complex displacements in the imaged plane were then extracted and Fourier transformed into the time domain, aligned with the experimental displacement data, and Fourier transformed back into the frequency domain for display of the real and imaginary parts in Fig. 3. Mesh adaptations were performed to allow refinements where the gradients of solutions were high. No mesh dependence was observed in final results.

## Results and discussion

### Experiment 1 and simulations

Figure 2 shows the distribution of the Lorentz force density along the *x*, *y*, and *z* axes in the imaging plane (Fig. 2). A significant contribution along the *x* axis was observed while contributions along the *y* and *z* axes were found negligible. This behavior is consistent given the expression of the Lorentz force density (vector cross-product) and main directions of current density (along *y* axis) and magnetic field (along *z* axis).

Figure 3 displays the experimentally measured displacement fields along the three directions of space and the simulated ones computed from the Lorentz force density distribution. The storage moduli used to characterize the phantom in these simulations were obtained from the mean values of the shear modulus distributions displayed in Fig. 4. The loss moduli were approximated to 1/10th of the storage moduli. Figure 3 shows the line profiles of the polarized displacement fields along the propagation directions for each tested combination of *G*’ and *G*" (Table 2).

In Fig. 3, the experimental displacement patterns along *x* and *z* allow for clear identification of the shear wave source, *i.e*. where the Lorentz force density is the highest (Fig. 2), namely between electrical contacts within the saline bath-phantom structure (Fig. 1a). No other shear wave origin could be observed, indicating the absence of movement artefacts. Experimental and simulated displacement fields show similar patterns. They both present a monopole-like arrangement along the *x* axis and a dipole mode along the *z* axis, at both frequencies. These patterns correspond to the shear mode described by the Green’s function solution to the inhomogeneous Navier’s equation^48^. However, no clear pattern could be identified along the *y* axis in experimental data where the motion amplitude was very low resulting in measurement errors (broad areas with negative-only or positive-only displacement values).

Simulated displacements along the *y* axis show maximum amplitudes on the order of 10 nm, which cannot be measured with the MR scanner. Although presenting the same modes and propagation directions as experimental data, simulated displacement fields feature wavelengths larger than measured ones, which suggests a mismatch in the definition of the material properties in the simulations rather than an underlying modeling issue. The storage moduli were assigned based on experimental estimations, which implies that the storage shear modulus distribution from the LFE inversion was overestimated. The line profiles along propagation directions for possible complex shear modulus values present the same behavior around the center of the line, which matches the distribution of the Lorentz force density, regardless of the mechanical parameters used. The side lobes towards opposite edges have, however, their own individual behavior. All simulated configurations provided maximum displacement amplitudes larger than the measured ones. This suggests that the experimental displacement amplitudes may have been underestimated, which would be consistent with their low values close to the reported detection limit of a few hundreds of nanometers in MRE^9^.

Potential global under evaluation of displacement amplitudes have theoretically no impact on the LFE reconstruction, which does not depend on the displacement amplitude but on the wavelength estimation so long as enough signal-to-noise ratio is available. Qualitatively, the noticeable change in wavelength between measurements at 60 Hz and 90 Hz further confirms the wave field dependence on the frequency of the Lorentz force density. The impact of current intensity on the displacement field was also observed in other experiments where the current intensity was varied. With a current at 60 Hz and 113 mA peak-to-peak, the mean norm of the displacement field calculated over the whole field of view was 0.14 µm. This value accordingly dropped to 0.07 µm when the current was lowered by a factor of 2. The same conclusion was drawn at 90 Hz with the displacement mean norm decreasing from 0.08 µm to 0.06 µm (ratio of 1.2) when the current was lowered from 149 mA peak-to-peak to 113 mA peak-to-peak (ratio of 1.3).

Figure 4 shows the shear storage modulus distributions obtained with the LFE inversion performed on *x* displacements. The LFE relies on the evaluation of the wave number in motion field images making it particularly sensitive to reflections off boundaries and wave interferences, which result in apparent larger or smaller wavelengths and consequently higher or lower shear moduli even in homogeneous tissues. This is assumed to be the case at 60 Hz and 90 Hz where regions with higher and lower values respectively formed semi-circle branches on the left- and right-hand sides. These arcs of circles are consistent with the wave front geometries of the experimental *x* displacements shown in Fig. 3. At the center of both frequency maps, higher shear moduli correspond to regions where the assumption of a body force free solid underlying the derivation of the elastic Helmholtz equation is no longer valid due to the presence of the Lorentz force density (Fig. 2). The presence of the Lorentz force density translates into overestimated shear moduli to compensate for the inertia term. The mean values over the entire field of views are *μ*_*60 Hz*_ = 1546 ± 383 Pa and *μ*_*90 Hz*_ $=1768\pm 440$ Pa.

### Experiment 2

**Figure 5:**
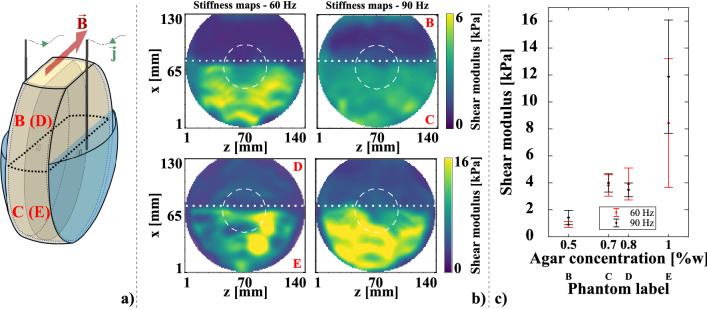
Second experiment. (**a**) Phantoms arrangement: B (D) on top of C (E). Phantom separation is indicated by the thick black dashed line. The greyed-out sagittal plane is the acquisition plane. (**b**) Stiffness maps from harmonic actuations at 60 Hz and 90 Hz with a current intensity of 62 mA peak-to-peak in phantoms B, C, D, and E. Mean values and standard deviations were calculated in compartments separated by the dotted line. The dashed circle indicates the region where the magnitude of the Lorentz force density drops from its maximum value to 1/10th of this maximum. (**c**) Mean shear storage moduli in phantoms B, C, D, and E. Error bars correspond to standard deviations. The separation between lower and higher parts of phantoms on stiffness maps, which corresponds to higher and lower agar concentrations, respectively, is clearly visible in panel (**b**).

Figure 5 displays results of the second experimental condition consisting in generating shear waves using the Lorentz force in phantoms with different mechanical properties. The shear modulus maps were generated from *x* displacements, as in Fig. 4, for the evaluation of the impact of the Lorentz force density on reconstruction (the Lorentz force density pulses along the *x* axis). Phantom names corresponding to upper and lower parts of the phantom compartments are indicated in red in upper and lower right corners of shear modulus maps. Despite the higher electrical resistance due to lower salt content in those phantoms, currents with sufficient intensities 62 mA peak-to-peak could be applied to conduct these measurements with sufficient signal-to-noise ratios.

The separation between the two phantom layers is clearly visible on all shear modulus maps. However, the distribution within each layer shows a significant variance, especially in the phantom E. No major difference was measured between phantoms C and D at both frequencies despite the difference in agar concentration. The dotted line shows the separation between the two compartments where the shear modulus mean values and standard deviations were calculated. The dashed circle in the middle represents the extent to which the Lorentz force varied from its maximum magnitude (centre of the circle) to 1/10th of this maximum (edge of the circle). No impact of the Lorentz force could be observed in this circle as opposed to the centre of shear modulus maps in Fig. 4. This may be explained by the spatial coincidence of the Lorentz force distribution and the stiffness discontinuity. The LFE was reported to lack sensitivity at the boundary between media of different stiffness^49^ because of the spatial extent of filters and the assumption of local homogeneity. This leads to estimated stiffness values to be inaccurate within a certain distance to media boundary.

Another impact of the Lorentz force on the resolution of the inverse problem may appear in the coupling of the shear (divergence free) and pressure (curl free) fields near the source, as opposed to the far field where shear and pressure waves travel at different speeds^50^. The pressure field was also shown in Ref.^13^ to introduce a DC component into the displacement field produced by external surface actuators (i.e., no body force and no coupling of shear and pressure fields). Applying the curl operator to the Navier’s equation allows to isolate the contribution of the shear field at the cost of noise enhancement due to third-order derivatives^13^. As aforementioned, the presence of a body force in the field of view of the imaged tissue theoretically leads to overestimated mechanical parameters where the source is. Additional mapping of the motion source distribution may then be necessary to correct for the overestimation. In other reported cases where body forces (other than Lorentz forces) are present, such as supersonic shear imaging^51^, conventional methods to discard the body force term in the Navier’s equation consist in selecting displacement data after the shear wave source was switched off or selecting ROIs far from the source. Chatelin et al*.*^52^ have developed a method to solve the inverse problem while taking body forces into account in the context of high intensity focused ultrasound. It allows to estimate optimal stiffness values through a minimisation process between experimental displacements and simulated displacements using *G*reen’s formalism. Such technique may provide the potential of including body forces in the calculation of the solution to the inverse problem in Lorentz force MR elastography.

Direct inversion techniques based on the Helmholtz equation conceptually allow for the addition of a source term in the formulation of the forward problem, but still rely on a local homogeneity assumption and high order derivatives. Identification techniques^14,53^ have shown good performance on brain MRE and involve the complete formulation of the viscoelastic forward problem, thus allowing for the addition of a source term. The gradient of mechanical parameters across the imaged domain is also taken into account. Additionally, such methods have been successful in configurations where the motion source, as in our case, is present in the field of view; that is in intrinsic MRE where the displacement field is produced by arteries pulsing within the imaged domain^28,30^.

In Lorentz force MRE, mapping the Lorentz force density distribution can be achieved using magnetic resonance electrical impedance tomography (MR-EIT), which allows to measure electrical current densities and conductivities of electrically inhomogeneous media^54^. Current in vivo techniques of MR-EIT resort to sets of electrodes attached to the tissue of interest^55^. Then, electric pulses are delivered to the tissue while being imaged, which presents many similarities with Lorentz force MRE. Additionally, MR-EIT is, like MRE, a phase contrast MR technique, which in turn may be combined using the Lorentz force as a tool for dual electrical–mechanical property mapping.

## Limitations

The present study investigated the feasibility of generating shear waves using a Lorentz force produced in the imaged phantom from the combination of the MR magnetic field and electrical stimulation. Despite successful generation and detection of motion fields, limitations are severalfold. In terms of MR sequence, phase difference images were output by the scanner but no access to raw data was possible, which limited detailed investigation of phase image pre-processing steps. Comparison of displacement fields measured with different fully accessible MR sequences using the same experimental setup would be necessary for quantitative assessment of Lorentz force MRE efficiency and to identify any MR sequence related variability. On the reconstruction end, the LFE performed well at producing images with clear delineations of global heterogeneous structures, which is key in medical imaging but showed significant variance within homogeneous structures in Fig. 5, and artefacts due to wave reflection and interference in Fig. 4. This makes further mechanical testing and cross-comparisons of shear modulus estimates using standard actuation systems meaningless at this stage. Particularly, accurate evaluation of the impact of the source term on the retrieved properties should be addressed in full development by comparing Lorentz force MRE to other MRE procedures, as those listed in the previous paragraphs, which is beyond the scope of the presented study. Finally, the main limitation is the amplitude of the electrical current that is for now around 30 times above the human security threshold (2 mA peak-to-peak in humans). Nevertheless, future studies should focus on implementing our method on pre-clinical MR scanners with higher magnetic fields and stronger encoding gradients, which would allow reducing significantly the intensity of the current necessary to have robust displacement maps. For specific human applications, implementation on 7 T or higher gradient scanners might be envisaged.

## Conclusion

In summary, this study demonstrated the feasibility of generating shear waves using the Lorentz force, producing stiffness maps from the displacement field, and reporting on potential impacts of the Lorentz force density on the reconstruction process. Overall, acquisitions were performed at two frequencies over three different phantom types implying removing and inserting phantom holders back into the experimental setup for each experimental condition. The consistent results obtained through multiple acquisitions indicate the robustness and reproducibility of the method. Despite significant variance in the shear modulus maps in Fig. 5, clear delineation of the two layers were observed, which is essential in medical imaging.

The LFE reconstructions could allow validating the working hypothesis to show the possibility of implementing the Lorentz force MRE method on a clinical scanner. This experimental condition using phantoms doped with physiological salt concentrations suggest that this method could be used for in vivo applications in MR elastography provided current intensities lower than 2 mA, as in transcranial alternative current stimulation (tACS) experiments^56,57^. This currently seems far from clinical applications but shows an interesting potential for preclinical studies where scanners with higher magnetic fields are available, thus producing a higher Lorentz force density for a given current value. Specific human applications on 7 T or higher magnetic field scanners might also be possible in the future.

Lowering the current intensity may also be reached by increasing other imaging parameters such as the motion encoding gradient amplitude (for instance to 285 mT m^−1^)^58^, the number of averaging over identical acquisitions repeated successively (to increase the signal-to-noise ratio), the number of ME*G* cycles (provided a suitable trade-off with the echo-time. Latitude thus remains to improve motion detection and reduce the current intensity. In terms of spatial selectivity, recent tACS results have shown that optimal electrode positioning on the scalp along with multi-frequency current application allow for delivering current to targeted deep regions into the brain^59^. Such current sequence design may be helpful in conceptualising Lorentz force MR brain elastography thus circumventing the low penetration of shear waves at high frequencies in conventional MR elastography due to significant attenuation. It may also provide the advantage of in situ localized vibrations to reduce diffraction artefacts by the skull when using an external vibration source.

As introduced above, a main advantage compared with conventional MR elastography is the fact that no external actuator is required thus excluding complex wave coupling between the external body and the imaged organ. The possibility of varying the shear wave frequency by a simple change in current frequency is another advantage. Eventually, Lorentz force MR elastography may be further developed as a remote mean to generate shear waves, notably with electric stimulation devices instead of electrodes, as in Ref. 18. At present, a framework involving tests of other MR sequences along with other reconstruction methods is currently being designed and may help sharpen the proposed technique. This will allow for quantitative characterization of the measured displacement fields using various encoding schemes and quantitative assessment of the impact of the Lorentz force density on the reconstructed mechanical parameter maps. The inclusion of MR-EIT protocols to Lorentz force MRE is also under development in an attempt to provide an original human or preclinical tool for assessment of both mechanical and electrical properties.
